# Methylphenidate-induced mania-like symptoms

**DOI:** 10.4103/0253-7613.75678

**Published:** 2011-02

**Authors:** Kaustav Chakraborty, Sandeep Grover

**Affiliations:** Department of Psychiatry, Institute of Neurosciences, Kolkata, 185/1, A.J.C. Bose Road, Kolkata - 700 017; India; 1 Department of Psychiatry, Post Graduate Institute of Medical Education and Research, Chandigarh - 160 012, India

**Keywords:** Adverse drug effect, attention deficit hyperactivity disorder, mental retardation, Methylphenidate, psychosis/mania

## Abstract

Therapeutic dose of methylphenidate is known to cause adverse effects (psychosis or mania), albeit in a small number of cases. Signs and symptoms of adverse effects usually disappear on stopping the medicine. Data regarding the safety of methylphenidate in comorbid attention deficit hyperactivity disorder (ADHD) and mental retardation are nonexistent. We describe a case of an 11-year-old girl with ADHD and mental retardation treated with methylphenidate, who developed mania like symptoms requiring inpatient treatment. The index case required psychopharmacological intervention with sodium valproate and olanzapine as the symptoms did not subside even after 3 days. This case highlights the fact that one has to exercise caution while prescribing methylphenidate in patients with comorbid ADHD and mental retardation.

## Introduction

The first report of stimulant-induced psychosis like or mania like symptoms in children included description of three cases, which the authors labeled as “methylphenidate hallucinosis”.[1] Since then, stimulants have been reported to induce euphoria, grandiosity, paranoid delusions, confusion, hallucinations, and increased aggression.[2,4] Over the years, the term “stimulant associated adverse drug effect” is used to describe the manic-like or psychotic like features associated with stimulants. In a review of data by Food and Drug Administration, “stimulant associated adverse drug effect” was reported to occur at a rate of 0.25%, i.e., an incidence of 1 in 400 children receiving stimulants in therapeutic doses.[5] Another review which included data from 49 randomized controlled trials found the rate of stimulant-induced psychosis/mania to be 1.48 per 100 person-years.[3] Although methylphenidate is in use in India for more than a decade, no case of adverse drug effect has been reported from India. Moreover, data regarding the safety of methylphenidate in patients of comorbid attention deficit/hyperactivity disorder (ADHD) and mental retardation are sparse.

We report a case of methylphenidate-induced mania like symptoms and discuss the risk factors associated with development of stimulant associated adverse drug effect.

## Case Report

An 11-year-old girl, born of full-term pregnancy which was complicated by prolonged labor, who had delayed speech, impairment in domains of communication, self-care, work, academic, interpersonal and social skills since early childhood presented with a history characterized by difficulties with sustained attention, distraction, poor compliance with instruction, loosing things, jumping and hopping around, not waiting for her turn during play, and interrupting parents when they would be interacting, since the age of 4 years. These symptoms increased over the years and were thought to be more than the appropriate mental age and led to marked dysfunction in social and school functioning. At the age of 6 years, she was diagnosed as having ADHD, combined type (as per DSM-IV), with moderate mental retardation (intelligence quotient of 46) at a tertiary care center and the parents were advised to follow the behavioral measures. There was no family or personal history suggestive of head injury, epilepsy, psychosis, affective disorders or substance abuse.

At the index assessment at the age of 11 years, the child fulfilled the diagnosis of ADHD, combined type (as per DSM-IV), with moderate mental retardation. Her body weight was 26 kg and physical examination did not reveal any abnormality. On investigation, her magnetic resonance imaging of brain, electroencephalogram, hemogram, serum electrolytes, renal function and liver function tests were found to be within normal limits. In view of the marked psychosocial dysfunction and failure to respond to behavioral measures in the past, she was prescribed methylphenidate 10 mg/day, which she tolerated well. After 1 week, the dose of methylphenidate was increased to 15 mg/day in divided doses. However, on the 4^th^ day of increase in dose, she was found to be restless (more than the past) with constant fidgeting, started running around, shouting, screaming and would bite family members if any attempt was made to calm her down. She spoke loudly at faster pace, hummed tunes and demanded for various food items and toys. However, she was able to recognize the parents and was aware of place and there was no history of abnormal involuntary movements. Also, she did not sleep throughout the night. Next day she was brought to the psychiatry OPD in a state of extreme agitation, running all over the places, opening tap, with socially disinhibited behavior. It was difficult to control her. There was no history of grandiosity, delusions or hallucinations. In view of the extreme agitation, she was admitted, methylphenidate was stopped and she was given Inj. lorazepam to control agitation. Her investigations were repeated including electroencephalogram and computerized tomography of brain, which were normal. For the initial 3 days, lorazepam was given in the dose of 4-8 mg/day intravenously, but there was no improvement in her symptoms. Following this, she was started on sodium valproate 600 mg/day in two divided doses along with olanzapine 5 mg/day. With these medications, by the 6^th^ day of admission, her symptoms started reducing and by the 9^th^ day, she improved significantly and was discharged on the 10^th^ day when she could interact in a meaningful way. Within a period of 1 month, both olanzapine and sodium valproate were withdrawn and the patient has been maintaining well.

## Discussion

Stimulant-induced adverse drug effect is an idiosyncratic reaction that cannot be predicted on the basis of age of the subjects, past history of psychiatric illness or family history, although it is advisable to exercise caution while using stimulant medications in children with familial affective disorders.[3,5,6] Stimulant-induced adverse drug effect is usually seen in children who premorbidly had attention deficit.[7,8] The index child had mental retardation along with attention deficit, which could possibly have predisposed the child to develop this adverse drug effect.

It is generally seen that stimulant-induced adverse drug effect is short lasting, with recovery typically occurring within 2 days and almost always within 7 days of discontinuing the medication or lowering the dose.[5] In the index child, the symptoms suggestive of adverse drug effect started after 3 days of increase in the dose of methylphenidate and did not resolve with stoppage of methylphenidate only and required the use of sodium valproate and olanzapine. Lack of resolution of adverse drug effect in the index case must be seen in the light of comorbid mental retardation, which could have contributed to the development and continuation of adverse drug effect.

It is suggested that, given the highly beneficial effects of stimulants for many patien ts with ADHD, rechallenge with a stimulant medication is often appropriate, although close follow-up is indicated.[9] In the index case, rechallenge was not attempted as parents were quite apprehensive and did not give consent for the same.

This case highlights the fact that therapeutic dose of methylphenidate can cause mania-like symptoms. Given the highly beneficial effect of methylphenidate in the treatment of ADHD, clinician should weigh the pros and cons before starting the medication. Moreover, because of relative lack of data on mentally retarded children, clinicians should exercise caution in prescribing this medication in these children.
